# Capillary tone: cyclooxygenase, shear stress, luminal glycocalyx, and hydraulic conductivity (*L*_p_)

**DOI:** 10.14814/phy2.12370

**Published:** 2015-04-20

**Authors:** Donna A Williams, Mary H Flood

**Affiliations:** Montana State UniversityBozeman, Montana

**Keywords:** Capillary physiology, mechanical force, microcirculation, water permeability

## Abstract

Control of capillary hydraulic conductivity (*L*_p_) is the physiological mechanism that underpins systemic hydration. Capillaries form the largest surface of endothelial cells in any species with a cardiovascular system and all capillaries are exposed to the flow-induced force, shear stress (*τ*). Vasoactive molecules such as prostacyclin (cyclooxygenase product, COX) are released from endothelial cells in response to *τ*. Little is known about how COX activity impacts capillary *L*_p_. The purpose here was to assess *L*_p_ in situ following an acute Δ*τ* stimulus and during COX1/COX2 inhibition. Mesenteric true capillaries (TC) of *Rana pipiens* (pithed) were cannulated for *L*_p_ assessment using the modified Landis technique. *Rana* were randomized into Control and Test groups. Two capillaries per animal were used (perfusate, 10 mg·mL^−1^ BSA/frog Ringer's; superfusate, frog Ringer's or indomethacin (10^−5^ mol·L^−1^) mixed in frog Ringer's solution). Three distinct responses of *L*_p_ to indomethacin (TC2) were demonstrated (TC1 and TC2 medians: Test Subgroup 1, 3.0 vs. 1.8; Test Subgroup 2, 18.2 vs. 2.2; Test Subgroup 3, 4.2 vs. 10.2 × 10^−7^ cm·sec^−1^·cm H_2_O^−1^). Multiple regression analysis revealed a relationship between capillary *L*_p_ and systemic red blood cell concentration or hematocrit, plasma protein concentration, and Δ*τ* (Test Subgroup 1, *R*^2^ = 0.59, *P *<* *0.0001; Test Subgroup 2, *R*^2^ = 0.96, *P *=* *0.002), but only during COX inhibition. Maintaining red blood cell and plasma protein levels within a normal range may control barrier function in a healthy state. Recovering barrier function may be an unrecognized benefit of transfusions during blood loss or edema formation.

## Introduction

Endothelial cells line the macro and microcirculatory components of the cardiovascular system and form the capillary network. As the primary cell type of capillaries, endothelial cells form the barrier between tissues and circulation that controls many processes in animals including hydration. As blood courses through the vascular system nutrients, oxygen, and water are delivered to all tissues and organs, and waste products are removed. Flow through the vascular network is required for the system to work efficiently as diffusion alone will not sustain life in multicellular species. Consequently, the cardiovascular lining and capillaries are exposed chronically to the mechanical forces shear stress and strain, which are necessary for continuous perfusion.

Two systemic variables, chronic shear stress (*τ*) and plasma proteins, are known to impact vascular permeability and capillary permeability. In vascular regions that are exposed chronically to low *τ*, high permeability is associated with atherosclerosis (Caro et al. 1971; Ku et al. 1985), an observation significant to the many investigations of cultured endothelial cells and *τ*. However, translating results from culture dish studies of *τ* to intact capillaries has yielded inconsistent results. Cultured endothelial cells increased permeability to sodium fluorescein, cyanocobalamin (Waters 1996), and hydraulic conductivity (*L*_p_; Jo et al. 1991; Kim et al. 2005; Sill et al. 1995) with increased flow-induced *τ*. Consistent with these observations, Williams (1999) studied intact capillaries and showed a positive relationship between *L*_p_ and acute change in shear stress (Δ*τ*). Similarly, Kajimura et al. (1998) reported increased K^+^ permeability with increased flow in intact capillaries. In contrast, Neal and Bates (2002) concluded that there was no relationship between *L*_p_ and *τ* for the intact capillaries in their study.

With regard to plasma proteins, Danielli (1940) reported edema formation in the frog hind limb when protein was removed from the perfusion solution. It has been suggested that whole organ permeability and single-capillary data are difficult to reconcile (Michel 1976). Of note, proteins did appear to affect fiber matrix (glycocalyx or endothelial surface layer) dimensions and account for differences between whole organ and single-capillary data, as well as species differences (Curry and Huxley 1982). Subsequent experiments on frog mesenteric capillaries and *L*_p_ have demonstrated a role for plasma proteins that is consistent with whole organ observations, reversible, and concentration dependent (Mason et al. 1977; Huxley and Curry 1985; Michel et al. 1985). However, despite the clear influence of protein on capillary *L*_p_, how, or if, systemic levels of plasma proteins correlate with capillary function has not been demonstrated.

The release of nitric oxide and prostacyclin from cultured endothelial cells and resistance microvessels is coupled (de Nucci et al. 1988; Koller et al. 1994) and occurs in response to *τ* (Frangos et al. 1985; Palmer et al. 1987). With regard to capillary *L*_p_, studies have demonstrated opposite conclusions with nitric oxide synthase (NOS) inhibition (Rumbaut et al. 1995; He et al. 1997). Moller and Grandë (1997) studied prostacyclin using isolated skeletal muscle tissue and showed increased permeability with indomethacin, a COX1/COX2 inhibitor. COX1/COX2 inhibition has not been tested in intact capillaries.

The purpose of this study was to test two hypotheses on intact capillaries following an acute Δ*τ* challenge: (1) *L*_p_ would increase during COX1/COX2 inhibition (indomethacin treatment) and reflect whole tissue results (Moller and Grandë 1997) and (2) *L*_p_ would be related to systemic variables known to impact endothelial cell function and permeability.

## Materials and Methods

Mesenteric capillaries of North American leopard frogs (*Rana pipiens*) were selected for their established history in the field of capillary physiology, the range of systemic chronic *τ*, and the quiescent in vivo tissue environment. The cage sedentary animals (*n* = 62, male, Alburg, VT and Nashville, TN) were housed 6 per container with access to fresh water and dry areas in an environmentally controlled room (15°C, 12 h:12 h light:dark cycle). Upon arriving at the animal care facility *Rana* were placed in a gentamicin (1.3 mg·mL^−1^) bath for the first 24 h. The animals were fed weekly and fasted for 4 days prior to the experiment. Fresh water was available ad libitum to prevent dehydration during fasting days. Animal Care and Use Committees at the University of Missouri, Columbia, MO and Montana State University, Bozeman, MT approved all animal handling procedures and protocols.

Surgical procedures have been published previously (Williams 1999, 2003, 2007, 2010). Briefly, each frog received a cerebral pith and cotton was placed in the cranial cavity to separate cerebral and spinal column tissue and minimize blood loss. Blood was sampled from a peripheral vein of the frog's right axilla, along the interior surface of the skin and exterior to the abdominal cavity. Care was taken to swab the area dry prior to blood collection to avoid diluting the sample. Lymph fluid was collected from the abdominal cavity for measurement of protein. Samples were drawn into heparinized microhematocrit tubes (74 *μ*L, not less than 2 USP units ammonium heparinase/tube; Fisher Scientific).

To prepare the mesentery for assessment of capillary function, a lateral incision was made through the skin and muscle wall. A loop of the small intestine was draped carefully over a quartz pillar and transilluminated. At the microscope, the tissue was kept moist and cool (14 to 16°C, type-T thermocouple wire, Digi-Sense®, Cole Parmer, Vernon Hills, IL) with fresh frog Ringer's solution superfused (≈3 mL·min^−1^) and siphoned away continuously. The mesenteric vasculature remained attached to the frog, intact, and blood-perfused during the entire protocol.

### Solutions

Frog Ringer's solution was prepared daily from 5× concentrated stock (pH 7.4 at 15°C) to the following (mM) concentrations: NaCl (111.0), KCl (2.4), MgSO_4_ (1.0), CaCl_2_ (1.1), glucose (5.0), NaHCO_3_ (2.0), and N-2-hydroxyethylpiperazine-N’-ethanesulfonic acid (HEPES)/Na-HEPES (5.0). Prior to experiments, bovine serum albumin (BSA, A-4378, Sigma Chemical Co., St. Louis, MO) was dialyzed (6000 to 8000 MWCO, Spectra/Por membrane, Spectrum, Houston, TX) and stored (1 mL aliquots, −20°C). On experiment day, an aliquot of BSA was thawed and dissolved in frog Ringer's (10 mg·mL^−1^ BSA). A small amount of human red blood cells (hRBC, 1 to 3% hematocrit) was added to the BSA solution to act as flow markers during assessment of capillary *L*_p_ (Williams 2007). This solution was kept on ice until loaded into the cannulation pipette. The same BSA lot number and human donor were used for all experiments to minimize variability. Indomethacin was dissolved in sodium carbonate (20 *μ*mol·L^−1^; Barber and Miller 1997) and diluted in frog Ringer's (10^−5^ mol·L^−1^).

### Systemic measures in blood and lymph fluid of *Rana pipiens*

#### Red blood cell concentration

A 5 *μ*L sample of whole blood was diluted 1:200 in filtered frog Ringer's solution (0.45 *μ*m pore size, Millipore, Billerica, MA) and loaded (10 *μ*L) into a Reichert bright line hemacytometer (Improved Neubauer ruling pattern, Cat. No. 1483, Hausser Scientific, Horsham, PA). Red blood cells were counted (Fisher Laboratory counter) using a Zeiss light microscope (Axiostar Plus, 10× A-plan objective). Red blood cells located completely within the ruling lines of the hemacytometer were included in the count. If a portion of a cell was outside the line it was not counted.

#### White blood cell concentration and activated white blood cell concentration

The same procedure as for red blood cell concentration (RBC) was followed to determine white blood cell concentration (WBC) with the only difference being a 1:20 dilution of a 12.5 *μ*L sample of whole blood. The diluted sample was loaded on the Neubauer slide and counted as for RBC.

To measure activated white blood cell concentration (aWBC), a solution of nitroblue tetrazolium dye was added to 12.5 *μ*L of whole blood, incubated at 37°C for 20 min and allowed to stand at room temperature for 20 min. The dye/whole blood sample was then diluted 1:20 with frog Ringer's and loaded on the Neubauer slide. The slide was inspected for aWBCs defined as those that had burst after taking up the dye according to Alexiou et al. (2004). The same inclusion decision rules were used as for RBC and WBC.

#### Hematocrit

Two blood-filled hematocrit tubes were double-sealed with critoseal (Fisher Scientific) and spun within minutes of blood collection at 13,460× g for 3 min (IEC Micro-MB Micro-centrifuge fitted with microhematocrit rotor). Hematocrit was measured using a microcapillary reader (IEC, Needham Heights, MA) with the indicator line placed at the interface of the red blood cell column and white blood cell layer. The reader was placed consistently at the same height and angle for each measurement. Intrasample reliability was within 1%.

#### Protein concentration

The plasma portion of the hematocrit sample and the lymph fluid samples were stored at −20°C for 2 months. A microassay (BioRad Laboratories, Inc., Hercules, CA) was used to measure protein concentration (Pr). According to manufacturer instructions (Bradford 1976). A standard curve and duplicate samples for each animal were read at 595 nm bandwidth (Turner SP-850 spectrophotometer, Barnstead/Thermolyne, Dubuque, IA).

#### Hemoglobin concentration

Hemoglobin concentration (Hb) was determined from a 20 *μ*L sample of whole blood mixed with 4.8 mL of Drabkin's Reagent (Sigma Chemical Co.) in a glass test tube. A standard curve was prepared with each assay using cyanomethemoglobin (range 20 to 80 mg·dL^−1^, Stanbio Diagnostics Co., Boerne, TX). One milliliter of the blood/Drabkin's mixture or standards solutions was pipetted into microcuvettes and read on a spectrophotometer (Turner) at a wavelength of 540 nm. Absorbance was recorded and Hb calculated from the standard curve. Mean corpuscular hemoglobin concentration (MCHb) was calculated as (Hb/Hematocrit) × 100.

### Individual capillary measures

#### Intravital video microscopy

Mesenteric capillaries were viewed with an inverted, compound microscope (UM 10× long working distance objective, 0.22 NA, Diavert, Leitz) and video recordings captured for each experiment (Panasonic AG-6300, Matsushita Electric Industries, JN) with time (0.01 sec, VTG-33, For-A, JN) superimposed onto the image (final magnification, 500×). Measurements were calibrated to a stage micrometer (0.01 mm, Meiji Techno, JN).

The microscope assembly and glass microtools used for cannulation of individual capillaries have been described previously (Williams 1999, 2003, 2007, 2010). A water manometer attached to the pipette assembly maintained pressure and flow.

#### Capillary identification

True capillaries (TC) were used exclusively and identified by direction of blood flow (divergent at one end and convergent at the other end, Chambers and Zweifach 1944). All TC were tubes of endothelial cells (≈1 *μ*m wall thickness) located centrally within the capillary network. TC were free of vascular smooth muscle, did not link arterioles directly to venules (not metarterioles), and had no rolling or sticking white blood cells.

#### Capillary tube hematocrit

Capillary tube hematocrit (tHct) was calculated from the frog red blood cell (fRBC) count per 500 *μ*m length of capillary and capillary radius (*r*, *μ*m) as:

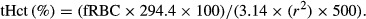
1

#### Capillary fRBC flux

Flux was assessed by selecting a point along the capillary segment then measuring the time (*t*, *s*) required for 50 red blood cells to pass that point. Flux was calculated as:

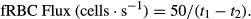
2

#### Capillary balance pressure

Upon cannulation, manometer pressure was lowered until the flow marker cells stopped. The balance or downstream pressure was recorded.

#### Capillary fluid shear rate (*γ*) and flow

Instantaneous RBC velocity (*ν*^i^) was measured directly on the video monitor at 15 sec intervals (Williams 1999, 2003, 2007, 2010):

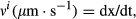
3

where *x* was distance (*μ*m) traveled by a RBC in time (*t*, *s*). Capillary diameter (*d*, *μ*m) was obtained from the average of three sites spaced ≈50 *μ*m apart on the video recording. Mean velocity (*ν*) was then calculated from *ν*^i^ and a correction factor (CF, hRBC radius = *R*, *μ*m and capillary radius, *d*/2 = *r*, *μ*m; Michel et al. 1974) assuming that each RBC was centered within the capillary:

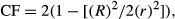
4

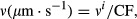
5

*γ* was calculated from *ν* for red blood cells and *r* for each capillary as:

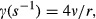
6

and capillary blood flow as:

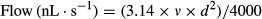
7

#### Estimated capillary fluid shear stress (*τ*)

The lower limit of apparent viscosity of blood (*η*^apparent^) was estimated from plasma protein (Chick and Lubrzynska 1914) and corrected to 15°C (Weast 1970; Williams 2007):

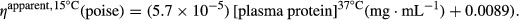
8

Capillary *τ* was estimated from *γ* and *η*^apparent^:

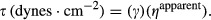
9

#### Capillary volume flux (*J*_v_) per surface area (*S*)

The modified Landis technique (Landis 1927; Michel et al. 1974) was used to measure *J*_v_ consistently at 30 cm H_2_O following a uniform, square wave Δ*τ* stimulus (see Experimental Protocol). Successful occlusion of each capillary was determined by visual inspection to insure valid measures of *J*_v_. *S* was calculated from radius (*r*, cm) assuming cylindrical geometry of capillaries. Three measures of diameter were obtained at 3 time points during each occlusion to verify that *S* remained constant.

hRBC marker cell velocity (dx/dt^occlusion^), capillary length (*x*_o_, cm), and the capillary volume to surface area ratio (*r*/2, cm) were measured for the first occlusion. *J*_v_/*S* calculated as:

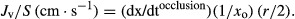
10

To increase precision, dx/dt^occlusion^ and *x*_o_ were measured on 3 hRBC (spaced ≈ 50 *μ*m apart) at three time points (2.0, 2.3, and 2.6 sec; Williams 1999, 2003, 2007, 2010).

#### Calculation of capillary *L*_p_ (cm·sec^−1^·cm H_2_O^−1^)

The Starling equation assumes a linear relationship between *J*_v_/*S* and capillary pressure (*P*_c_). The slope of the regression equation for *J*_v_/*S* and *P*_c_ is *L*_p_:

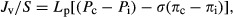
11

where (*P*_c_ − *P*_i_) and (*π*_c_ − *π*_i_) are hydrostatic (*P*) and oncotic (*π*) pressure differences between capillary lumen (c) and interstitium (i). Sigma (*σ*) is the reflection coefficient of the capillary wall to protein (for assumptions see Williams 2007). For this study, capillary *L*_p_ was calculated from nine measures of *J*_v_/*S* obtained during the first occlusion at 30 cm H_2_O (see above) and averaged. Under circumstances where a rapid change in flux occurs we, and others (Neal and Michel 1995), have used zero as the *x*-axis intercept (*σ*(*π*_c_ − *π*_i_)), a procedure that conservatively underestimates *L*_p_ (Williams 1999, 2003, 2007, 2010).

### Fluid mechanical stimulation of individual capillaries and experimental protocol

#### Fluid shear stress stimulus

Each capillary was cannulated at 10 cm H_2_O (7.4 mm Hg), balance pressure was measured, and low flow was established for a 2-min equilibration period (Steady-State 1). At the end of Steady-State 1, capillary perfusion pressure was changed abruptly to 30 cm H_2_O (22.1 mm Hg) to produce a uniform, acute, square-wave change in shear stress (Δ*τ*). The new, higher level of *τ* was maintained for 2 min, Steady-State 2, and then *J*_v_/*S* was measured (see above). Magnitude of the fluid mechanical stimulus (Δ*τ*) was calculated as:

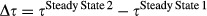
12

A physiological range for Δ*τ* occurred in accordance with the downstream resistance of the microcirculation for each animal. Video recordings verified the square wave stimulus and steady-state plateaus. In general, it was assumed that filtration during occlusion reflected filtration at Steady-State 2. Important advantages were that each capillary remained undisturbed downstream and the Δ*τ* stimulus was applied uniformly to each capillary (Williams 1999, 2003, 2007, 2010).

#### Protocol

Animals were randomized into Control group and Test group. Two mesenteric capillaries per animal (TC1 and TC2) were cannulated in series and each stimulated with Δ*τ* (see above). TC1 and TC2 in the Control group animals were perfused with BSA in frog Ringer's solution and superfused with frog Ringer's solution during *L*_p_ assessments. TC1 and TC2 in the Test group animals were perfused with BSA in frog Ringer's. TC1 (internal control) was superfused with frog Ringer's and TC2 (experimental capillary) was superfused with indomethacin (10^−5^ mol·L^−1^, 5 min) during *L*_p_ assessment. A few Test group TC2 capillaries were perfused (rather than superfused) with indomethacin (10 mg·mL^−1^ BSA/frog Ringer's) to control for any tissue effects with indomethacin superfusion.

To minimize variability, the PI performed all experimental protocols, collected body fluid samples, and measured hematocrit. The laboratory technician measured all other systemic and capillary variables (listed in Table1) to minimize measurement error and bias. Data were collected in all months minus October due to scheduling conflicts.

**Table 1:** Control group and test subgroup mean (± SD) values for systemic and capillary variables measured in *Rana pipiens* (*n*).

	Control Group (11)	Test Subgroup 1 (20)	Test Subgroup 2 (8)	Test Subgroup 3 (8)
Systemic Variables				
Weight (gm)	29.0 ± 4.5	29.6 ± 5.8	32.6 ± 6.0	31.3 ± 6.2
Length (cm)	18.8 ± 0.5	18.7 ± 1.1	19.1 ± 1.4	19.1 ± 0.9
Heart Rate (bpm) (pithed)	54 ± 15	66 ± 8	64 ± 12	62 ± 7
RBC × 10^3^ (cells·cm^−3^)	319 ± 66	345 ± 121	213 ± 45^1^	275 ± 89
Hematocrit (%)	31.3 ± 9.2	27.3 ± 8.7	20.6 ± 5.9	25.3 ± 6.4
Hb (g·dL^−1^)	7.2 ± 1.3	8.0 ± 2.1	6.1 ± 0.9	6.6 ± 2.0
MCHb	24.4 ± 6.0	31.2 ± 9.9	31.6 ± 10.3	26.2 ± 3.7
Plasma Pr (mg·mL^−1^)	20.5 ± 8.9	23.9 ± 7.5	23.5 ± 6.7	25.2 ± 7.0
Abdominal Cavity Pr (mg·mL^−1^)	6.4 ± 1.5	6.9 ± 2.5	6.3 ± 3.2	6.1 ± 3.0
WBC (cells·cm^−3^)	5990 ± 2857	3588 ± 1259	3800 ± 1742	4988 ± 1835
Capillary Variables				
TC1 *L*_p_ × 10^−7^ (cm·sec^−1^·cm H_2_O^−1^)	3.1 ± 1.7	3.2 ± 1.7	22.8 ± 12.1^2^	4.6 ± 2.5
TC2 *L*_p_ × 10^−7^	4.4 ± 2.6	2.3 ± 1.2	3.0 ± 1.7	15.9 ± 11.2^3^
Length (mm)	1.2 ± 0.3	1.2 ± 0.5	1.1 ± 0.5	1.2 ± 0.4
In situ Diameter (*μ*m)	12.6 ± 1.3	13.4 ± 2.0	12.6 ± 3.4	13.7 ± 3.8
Δ Diameter (*μ*m)	3.2 ± 2.7	0.9 ± 2.3	3.6 ± 3.1^4^	1.9 ± 2.7
Mean Velocity (*μ*m·sec^−1^)	714.4 ± 549.7	891.6 ± 1068.4	561.7 ± 420.6	762.6 ± 378.5
Shear Rate (sec^−1^)	442.4 ± 319.9	530.5 ± 625.4	381.3 ± 295.1	446.6 ± 190.1
Estimated *τ* (dynes·cm^−2^)	7.3 ± 5.5	8.9 ± 10.6	6.3 ± 4.8	7.5 ± 3.0
Δ*τ* (dynes·cm^−2^)	34.3 ± 13.6	37.2 ± 12.8	38.5 ± 22.9	34.7 ± 8.5
Balance Pressure (cm H_2_O)	8.9 ± 3.2	10.5 ± 2.3	10.4 ± 3.0	10.8 ± 3.1
Tube Hematocrit (%)	12.4 ± 5.9	9.0 ± 6.1	10.5 ± 8.6	8.9 ± 3.9
Flux (cells·sec^−1^)	51.5 ± 39.4	46.6 ± 36.2	48.7 ± 52.1	45.7 ± 30.7
Flow (nL·sec^−1^)	97.0 ± 85.2	137.4 ± 178.2	70.0 ± 66.2	135.6 ± 103.0

RBC, red blood cell concentration; Hb, hemoglobin; MCHb, mean corpuscular hemoglobin; Pr, protein; WBC, white blood cell concentration; TC, true capillary; Lp, hydraulic conductivity; Δ*τ*, change in shear stress.

1 Test Subgroup 2 < 1, *P *=* *0.01.

2 Test Subgroup 2 > 1 and 3, *P *<* *0.0001.

3 Test Subgroup 3 > 1 and 2, *P *<* *0.0001.

4 Test Subgroup 2 > 1, *P *=* *0.05.

### Statistical analyses

Normality of each data set was assessed using a Shapiro–Wilk W test and central tendency of the data are reported as mean (±SD) or median (±25% quartile and 75% quartile) as appropriate. For all analyses, “n” equals the number of animals. Box plots and nonparametric density were used to identify outliers. Student's *t*-tests or Wilcoxon signed-rank tests were used to determine differences between means or medians, respectively. Linear regression and multiple regression analyses were used to assess relationships between capillary *L*_p_ and one or more independent variables, respectively (JMP software, SAS Institute, Inc., Cary, NC). The highest *R*^2^ value was used to determine best fit. Significance was set at *P *≤* *0.05 during the design phase of the study.

## Results

All *Rana pipiens* used in this study were deemed healthy by inspection. Average (±SD) weight was 29.0 ± 4.5 and 30.6 ± 5.9 gm for the Control (*n* = 11) and Test (*n* = 36) groups, respectively. All *Rana* tested negative for aWBC and were devoid of the lung parasite, *Haematoloechus* sp. The most common configuration on the video monitor was a single, mesenteric capillary pictured with no others visible. Capillaries averaged (±SD) 1.3 ± 0.5 mm in length and 13.5 ± 2.4 *μ*m in diameter.

### *L*_p_ of two true capillaries per tissue

**Figure 1:**
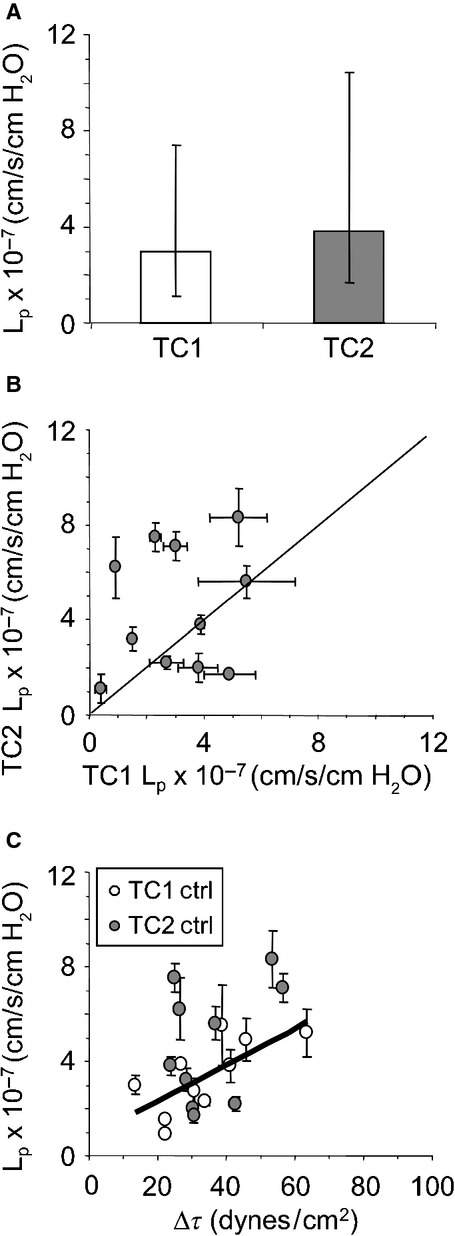
Hydraulic conductivity (*L*_p_) assessed on capillary pairs (TC1 and TC2) located in mesenteric tissue of Control group *Rana pipiens* and following acute change in shear stress (Δ*τ*). TC1 and TC2 were superfused with frog Ringer's solution and perfused with BSA/frog Ringer's solution during *L*_p_ assessments. (A) Median *L*_p_ (±25 and 75%) for TC1 and TC2 (*n* = 11 capillary pairs), *P *=* *0.24 (Wilcoxon). (B) Individual values of *L*_p_ (±SD) for TC1 and TC2. Each data point is one animal, solid line = line of identity. (C) *L*_p_ (±SD) plotted as a function of Δ*τ*. Solid line: TC1, *L*_p_ = 0.74 + 0.08(Δ*τ*), *R*^2^ = 0.50, *P *=* *0.02; TC2, no relationship between *L*_p_ and Δ*τ*, *P *=* *0.25. One TC1, Δ*τ* outlier along with its TC2 pair was removed from the *L*_p_/Δ*τ* analysis.

Figure1 illustrates median *L*_p_ and individual data points for Control group capillaries. Both data sets were distributed normally (*P *>* *0.05) and neither mean nor median *L*_p_ differed for TC1 compared to TC2 (Fig.1A). Individual data points for TC1 *L*_p_ versus TC2 *L*_p_ clustered uniformly around the line of identity (Fig.1B) and magnitude of acute Δ*τ* was related to TC1 *L*_p_, but not to TC2 *L*_p_ (Fig.1C). Table1 contains averages for systemic and capillary variables in the Control group.

### Indomethacin and *L*_p_ of true capillaries

**Figure 2:**
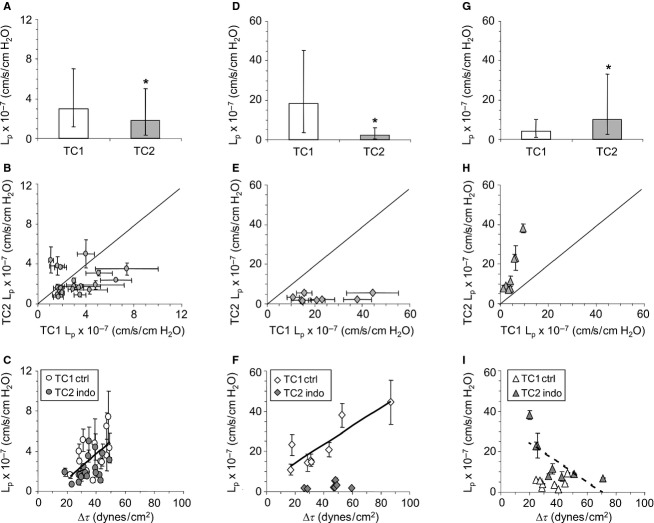
Hydraulic conductivity (*L*_p_) assessed on capillary pairs (TC1 and TC2) located in mesenteric tissue of the Test subgroups of *Rana pipiens* and following acute change in shear stress (Δ*τ*). Capillaries were superfused with frog Ringer's (TC1) or indomethacin (10^−5^ mol·L^−1^) mixed in frog Ringer's (TC2). (A) Test Subgroup 1: median *L*_p_ (±25 and 75%) for TC1 and TC2 (*n* = 20 capillary pairs), *P =* *0.05. (B) Test Subgroup 1: individual values of *L*_p_ (±SD) for TC1 and TC2. Each data point is one animal, solid line = line of identity. (C) Test Subgroup 1: *L*_p_ (±SD) plotted as a function of Δ*τ*. Solid line: TC1, *L*_p_ = −1.3 + 0.13(Δ*τ*), *R*^2^ = 0.35, *P *=* *0.01; TC2, no relationship between *L*_p_ and Δ*τ*, *P *=* *0.12. Two TC1 Δ*τ* outliers and one TC2 Δ*τ* outlier along with their pair were removed from the *L*_p_/Δ*τ* analysis. (D) Test Subgroup 2: median *L*_p_ (±25 and 75%) for TC1 and TC2 (*n* = 8 capillary pairs), *P =* *0.0004. (E) Test Subgroup 2: individual values of *L*_p_ (±SD) for TC1 and TC2. Each data point is one animal, solid line = line of identity. (F). Test Subgroup 2: *L*_p_ (±SD) plotted as a function of Δ*τ*. Solid line: TC1, *L*_p_ = 5.0 + 0.46(Δ*τ*), *R*^2^ = 0.76, *P *=* *0.005; TC2, no relationship between *L*_p_ and Δ*τ*, *P *=* *0.47. (G) Test Subgroup 3: median *L*_p_ (±25 and 75%) for TC1 and TC2 (*n* = 8 capillary pairs), *P =* *0.003. (H) Test Subgroup 3: individual values of *L*_p_ (±SD) for TC1 and TC2. Each data point is one animal, solid line = line of identity. (I) Test Subgroup 3: *L*_p_ (±SD) plotted as a function of Δ*τ*. TC1, no *L*_p_/Δ*τ* relationship, *P *=* *0.71. Dashed line: TC2, *L*_p_ = 34.4 − 0.49(Δ*τ*), *R*^2^ = 0.53, *P *=* *0.04. Note differences in ordinate scales.

*L*_p_ data were merged for capillaries perfused versus superfused with indomethacin as no differences could be detected between the two methods of drug delivery. *L*_p_ data for all TC1 capillaries in the Test group were not distributed normally (*P *<* *0.0001, median 4.1 × 10^−7^, mean ±SD 7.9 ± 9.9 × 10^−7^ cm·sec^−1^·cm H_2_O^−1^). Similarly, *L*_p_ data for all TC2, indomethacin-treated capillaries were skewed left (*P *<* *0.0001) suggesting a mixed sample of either capillaries or animals (median 2.4 × 10^−7^, mean ± SD 5.4 ± 7.6 × 10^−7^ cm·sec^−1^·cm H_2_O^−1^). Median TC2 *L*_p_ (indomethacin) did not differ from median TC1 *L*_p_ (*P *=* *0.07, Wilcoxon). In general, *L*_p_ data showed more stability during assessment (*y*-axis error bars < *x*-axis error bars) when indomethacin was present. From this analysis alone, hypothesis 1 was not supported; however, outlier analysis of the *L*_p_ data revealed clusters of data large enough to be retained as three distinct subgroups (Fig.2).

Test Subgroup 1 was comprised of the capillaries/animals separated from the outliers. Test Subgroup 2 was the TC1 outliers plus each TC2 pair. Test Subgroup 3 was the TC2 outliers plus each TC1 pair. Median *L*_p_ for Test Subgroup 1 TC2 capillaries (indomethacin) was slightly lower than for the control (TC1) pairs (Fig.2A). For Test Subgroup 2, however, median *L*_p_ for TC2 (indomethacin) was seven to eightfold lower compared to TC1 pairs (Fig.2D). In contrast, median *L*_p_ for TC2 in Test Subgroup 3 was almost fourfold higher (rather than lower as for Test Subgroups 1 and 2) with indomethacin relative to that for the TC1 pairs (Fig.2G). The result from Test Subgroup 3 supports hypothesis 1.

Table1 provides averages for systemic and capillary variables measured in Test Subgroups 1, 2, and 3. All systemic variables were similar between the three test subgroups with one exception, RBC, which was lower for Test Subgroup 2. Test Subgroup 2 also displayed the highest values for control *L*_p_ and the greatest decrease in *L*_p_ with indomethacin. For capillary variables, averages were similar between the three test subgroups with the exception of Δ diameter, which was greater for Test Subgroup 2 capillaries. Δ diameter was similar for Control group and Test Subgroup 2 capillaries suggesting that Δ diameter did not account for the changes in *L*_p_ with indomethacin displayed in Test Subgroup 2. Additionally, changes in capillary balance pressure in response to indomethacin did not correlate with changes in capillary *L*_p_ for any of the test subgroups suggesting that microvessel responses to COX1/COX2 inhibition were not regulated synchronously with capillary responses (data not shown).

### Relationship between capillary *L*_p_ and Δ*τ*

*L*_p_ data for the three test subgroups were analyzed relative to magnitude of the acute Δ*τ* challenge. For Test Subgroup 1, TC1, *L*_p_ was related to Δ*τ*; however, no *L*_p_/Δ*τ* relationship was identified for TC2 (indomethacin, Fig.2C). Similarly, Test Subgroup 2 TC1 *L*_p_ displayed a strong-positive relationship with Δ*τ* and no *L*_p_/Δ*τ* relationship for TC2 (indomethacin, Fig.2F). Test Subgroup 3 TC1 *L*_p_ data were not related to Δ*τ*; however, TC2 *L*_p_ values (indomethacin) did display a significant relationship with Δ*τ* (Fig.2I), a result that contrasted with Test Subgroups 1 and 2.

### Predictors of capillary *L*_p_

**Figure 3:**
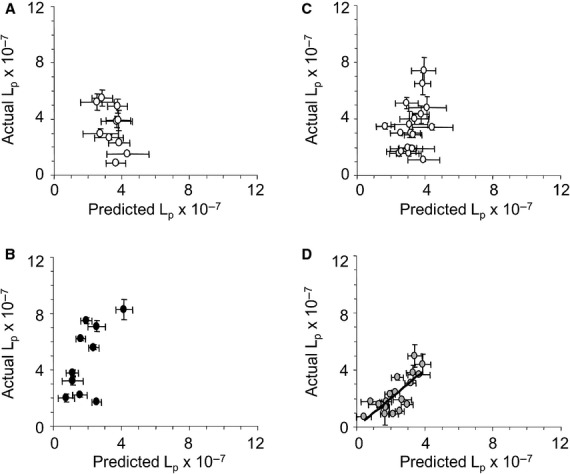
Predicted versus actual assessments of hydraulic conductivity (*L*_p_ ± SD × 10^−7^ cm·sec^−1^·cm H_2_O^−1^) following acute change in shear stress (Δ*τ*) stimulation of capillaries located in mesenteric tissue of Control group and Test Subgroup 1 *Rana pipiens*. Red blood cell concentration, plasma protein concentration, and change in shear stress (Δ*τ*) were used in the multiple regression analysis to predict *L*_p_. (A) Control group TC1 capillaries: superfused with frog Ringer's and perfused with BSA/frog Ringer's solutions. (B) Control group TC2 capillaries: superfused with frog Ringer's and perfused with BSA/frog Ringer's solutions. (C) Test Subgroup 1 TC1 capillaries: superfused with frog Ringer's and perfused with BSA/frog Ringer's solutions. (D) Test Subgroup 1 TC2 capillaries: superfused with indomethacin (10^−5^ mol·L^−1^, 5 min) mixed in BSA/frog Ringer's and perfused with BSA/frog Ringer's solutions. Solid line: significant relationship (*R*^2^ = 0.59, *P *<* *0.0001) between actual versus predicted values of capillary *L*_p_ (model estimates provided in Table3).

Results from multiple regression analysis supported the hypothesis that systemic variables associated with chronic τ (RBC or hematocrit) and the luminal glycocalyx (plasma protein) combined with Δ*τ* to predict capillary *L*_p_. Figure3 shows the results of predicted versus actual *L*_p_ values for the Control group and Test Subgroup 1. No relationships were found for Control group TC1, Control group TC2, and Test Subgroup 1 TC1 (Fig.3A–C) using systemic RBC, systemic plasma protein, and Δ*τ* as the independent variables. However, those same three independent variables in the model did predict *L*_p_ for Test Subgroup 1 TC2 (indomethacin) (Fig.3D). Table3 contains the model estimates.

**Figure 4:**
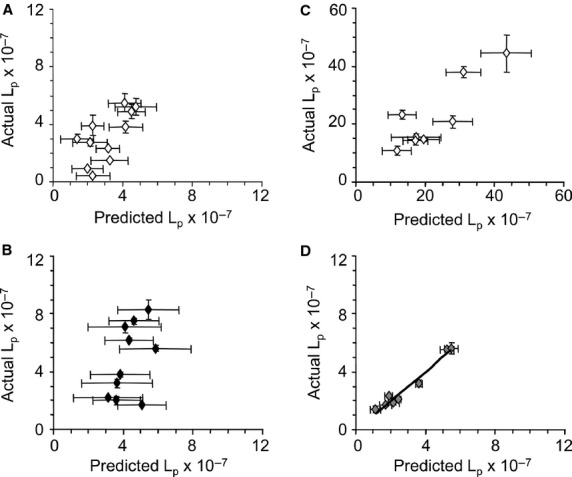
Predicted versus actual assessments of hydraulic conductivity (*L*_p_ ± SD × 10^−7^ cm·sec^−1^·cm H_2_O^−1^) following acute change in shear stress (Δ*τ*) stimulation of capillaries located in mesenteric tissue of Control group and Test Subgroup 2 *Rana pipiens*. Hematocrit, plasma protein concentration, and Δ*τ* were used in the multiple regression analysis to predict *L*_p_. (A) Control group TC1 capillaries: superfused with frog Ringer's and perfused with BSA/frog Ringer's solutions. (B) Control group TC2 capillaries: superfused with frog Ringer's and perfused with BSA/frog Ringer's solutions. (C) Test Subgroup 2 TC1 capillaries: superfused with frog Ringer's and perfused with BSA/frog Ringer's solutions. (D) Test Subgroup 2 TC2 capillaries: superfused with indomethacin (10^−5^ mol·L^−1^, 5 min) mixed in BSA/frog Ringer's and perfused with BSA/frog Ringer's solutions. Solid line: significant relationship (*R*^2^ = 0.96, *P *=* *0.002) between actual versus predicted values of capillary *L*_p_ (model estimates provided in Table3). Note differences in ordinate scales.

Data for capillaries in Control group and Test Subgroup 2 are presented in Figure4. Similar to Test Subgroup 1, significant prediction of *L*_p_ was achieved only for the indomethacin-treated capillaries (Fig.4D). An important distinction, however, was that systemic hematocrit (not RBC) was the index for chronic *τ* that produced the strongest model. Table3 contains model estimates for predicting *L*_p_ in Test Subgroup 2 TC2. No predictors of capillary *L*_p_ were identified for Test Subgroup 3 capillaries.

### Prospective prediction of capillary *L*_p_

**Figure 5:**
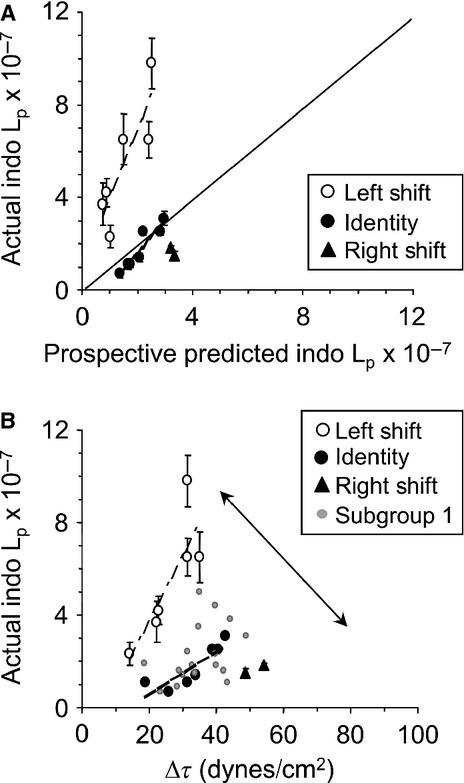
Prospective evaluation of prediction model for Test Subgroup 1 capillary hydraulic conductivity (*L*_p_ ± SD × 10^−7^ cm·sec^−1^·cm H_2_O^−1^) with indomethacin. (A) Predicted *L*_p_ versus actual *L*_p_ assessed on capillaries during indomethacin treatment where three subsets, Left Shift (open circles, dot-dash line), Identity (black closed circles, dashed line), and Right Shift (closed triangles) were identified. Solid line = line of identity (B) Actual TC2 *L*_p_ (indomethacin) plotted as a function of change in shear stress (Δ*τ*). Left Shift subset: *L*_p_ = −1.9 + 0.28(Δ*τ*), *R*^2^ = 0.69, *P *=* *0.04; Identity subset: *L*_p_ = −1.3 + 0.09(Δ*τ*), *R*^2^ = 0.75, *P *=* *0.01. Original data from Test Subgroup 1 (Figure2C, gray closed circles) have been added for comparison. Double arrowhead solid line: span of capillary *L*_p_ tone.

The same protocol was followed on 15 additional animals/capillaries to test prospectively the prediction model derived from Test Subgroup 1 (Table3). The second set of animals averaged (±SD) 34.8 ± 8.8 gm. Key predictors (systemic RBC, systemic plasma protein, and Δ*τ*) were entered into the model and TC2 *L*_p_ (indomethacin) was calculated. Next, *L*_p_ was assessed on two capillaries per animal with TC2 receiving indomethacin treatment as above. The predicted versus actual *L*_p_ values are presented in Figure5A. Although all *L*_p_ values fell within the range of Test Subgroup 1, unexpectedly, these capillaries/animals separated into three subsets. Regression analysis indicated that the calculated and actual *L*_p_ values displayed strong relationships in Left Shift and Identity subsets. Too few data points in the Right Shift data prevented further analysis.

Figure5B shows the actual TC2 *L*_p_ data as a function of Δ*τ*. Data from Test Subgroup 1 were added to the figure for comparison and context. The regression line calculated for the Identity subset was very close to that for the original data in Test Subgroup 1. The Left Shift and Right Shift subsets bracketed the limits of the *L*_p_/Δ*τ* response illustrating a pattern or spectrum for capillary *L*_p_ tone that widened as Δ*τ* and *L*_p_ increased. Table2 contains averages for systemic and capillary variables for the Left Shift, Identity, and Right Shift animals/capillaries. All systemic variables and capillary variables were similar between the three subsets of animals.

**Table 2:** Mean (±SD) values for systemic and capillary variables measured in *Rana pipiens* (*n*) and sorted into the subsets identified from prospective prediction model for *L*_p_ with indomethacin.

	Left Shift (6)	Identity (7)	Right Shift (2)
Systemic Variables			
Weight (gm)	35.3 ± 9.4	33.2 ± 9.2	39.1 ± 8.8
Length (cm)	19.9 ± 0.5	19.3 ± 1.2	20.5 ± 1.6
Heart Rate (bpm) (pithed)	53 ± 9	57 ± 5	64 ± 1
RBC × 10^3^ (cells·cm^−3^)	316 ± 79	383 ± 41	454 ± 93
Hematocrit (%)	26.2 ± 4.4	32.2 ± 8.5	33.4 ± 1.7
Hb (g·dL^−1^)	9.1 ± 1.9	9.9 ± 2.5	10.0 ± 0.4
MCHb	34.4 ± 2.3	31.3 ± 5.5	30.0 ± 2.6
Plasma Pr (mg·mL^−1^)	23.6 ± 4.6	28.4 ± 5.2	29.0 ± 0.8
Abdominal Cavity Pr (mg·mL^−1^)	4.7 ± 0.5	4.3 ± 1.0	nd
WBC (cells·cm^−3^)	2717 ± 549	2779 ± 547	3400 ± 141
Capillary Variables			
TC1 *L*_p_ × 10^−7^ (cm·sec^−1^·cm H_2_O^−1^)	5.3 ± 3.2	3.6 ± 2.2	4.5 ± 4.2
TC2 *L*_p_ × 10^−7^ (Indomethacin)	5.5 ± 2.7^1^	1.8 ± 0.9	1.7 ± 0.2
Length (mm)	1.4 ± 0.9	1.6 ± 0.9	1.4 ± 0.7
In situ Diameter (*μ*m)	14.9 ± 3.3	13.4 ± 2.9	11.8 ± 0.7
Δ Diameter (*μ*m)	2.2 ± 1.7	1.7 ± 2.7	3.1 ± 0.5
Mean Velocity (*μ*m·sec^−1^)	592.7 ± 380.0	582.9 ± 338.5	257.5 ± 50.2
Shear Rate (sec^−1^)	302.7 ± 187.0	361.5 ± 233.7	173.3 ± 23.7
Estimated *τ* (dynes·cm^−2^)	5.1 ± 3.2	6.2 ± 4.0	3.0 ± 0.4
Δ*τ* (dynes·cm^−2^)	27.4 ± 11.4	27.3 ± 8.9	38.1 ± 10.0
Balance Pressure (cm H_2_O)	11.7 ± 3.6	11.4 ± 4.2	12.9 ± 2.7
Tube Hematocrit (%)	8.7 ± 5.0	10.0 ± 4.8	nd
Flux (cells·sec^−1^)	64.0 ± 16.1	40.8 ± 29.0	18.8 ± 21.3
Flow (nL·sec^−1^)	121.4 ± 86.6	85.0 ± 54.2	28.5 ± 8.9

Capillary comparisons: ^1^Left Shift > Identity, *P *=* *0.005. RBC, red blood cell concentration; Hb, hemoglobin; MCHb, mean corpuscular hemoglobin; Pr, protein; WBC, white blood cell concentration; TC, true capillary; Lp, hydraulic conductivity; Δ*τ*, change in shear stress.

## Discussion

The purpose of this study was to assess *L*_p_ of living capillaries following acute Δ*τ* and during COX1/COX2 inhibition (indomethacin). *L*_p_ was evaluated relative to Δ*τ* and systemic variables known to impact endothelial cell function and permeability. Detailed analyses revealed three test subgroups of capillaries/animals. COX1/COX2 inhibition decreased *L*_p_ in the first two and increased *L*_p_ in the third. *L*_p_ was predicted from RBC or hematocrit, plasma protein, and Δ*τ*, but only for capillaries treated with indomethacin. A relationship between systemic variables and capillary function measured in the same animals has not been demonstrated previously.

### Acute Δ*τ* stimulation of intact capillaries

All capillaries in the original data set were homogeneous based on selection criteria, including visual cues of flow direction (strict definition of TC) and no rolling or sticking WBC. Despite these carefully adhered to criteria, frequency distributions of capillary function (TC1 *L*_p_ and TC2 *L*_p_) revealed left skew suggesting a mixed sample of capillaries/animals and, in fact, extreme outliers were identified. The relatively high number of statistical outliers suggested that valuable information would be lost if these data were removed entirely from the analysis, so the decision was made to retain the data in three separate test subgroups, possibly as samples from three populations.

*L*_p_/Δ*τ* scatter plots proved useful to further support the existence of three samples of capillaries/animals. TC1 in the Control group, Test Subgroup 1, and Test Subgroup 2 all displayed positive and significant *L*_p_/Δ*τ* relationships, consistent with data published previously on intact capillaries (Kajimura et al. 1998; Williams 1999, 2003, 2007, 2010) and cultured endothelial cells (Jo et al. 1991; Sill et al. 1995; Kim et al. 2005). For Test Subgroup 1 (Fig.2C), 35% of the variability in TC1 *L*_p_ was accounted for by Δ*τ* alone. In contrast, Δ*τ* accounted for 75% (threefold larger percentage) of the variability in *L*_p_ for the TC1 capillaries in Test Subgroup 2 (Fig.2F) suggesting a different subcellular milieu. Absence of a significant *L*_p_/Δ*τ* relationship for TC1 in Test Subgroup 3 (Fig.2I) also was consistent with a different basal state. Collectively, these analyses did support three test subgroups, each sampled from a different population. The analyses also illustrated how *L*_p_/Δ*τ* can distinguish differences in capillary function that may reflect hydration-related “micro”-differences between animals that are not detectable via “macro” (clinical)-inspection.

Neal and Bates (2002) embarked on an extensive analysis of *τ* and *L*_p_ employing a double-cannulation method. We approximated values for *τ* and *L*_p_ from a scatter plot published by Neal and Bates (2002; Figure 13) and, using nonparametric-density analysis, identified two very clear outliers in their data set. Regression analysis performed on the seven remaining data points revealed a strong and significant *L*_p_/Δ*τ* relationship: *R*^2^ = 0.89, *P *=* *0.002. Although we do not have the precise values, the approximated values provided an impressive confirmation of the results reported here for TC1 capillaries and in the past (Kajimura et al. 1998; Williams 1999, 2003, 2007, 2010). Additionally, the *L*_p_/Δ*τ* slope of the Neal and Bates’ data was approximately 0.39, impressively close to the value of 0.46 for Test Subgroup 2. Note: our conclusion is opposite to that of Neal and Bates.

### COX1/COX2 inhibition and acute Δ*τ*

The decrease in TC2 *L*_p_ with indomethacin for Test Subgroups 1 and 2 indicated that COX inhibition had protected the barrier from acute Δ*τ*. These results clearly did not support hypothesis 1. The large decrease in *L*_p_ with indomethacin displayed by TC2 in Test Subgroup 2 suggested that COX1/COX2 activity was elevated in these capillaries. As such, we hypothesize that COX1/COX2 products in Test Subgroup 2 exerted considerable control over the response of capillaries to acute Δ*τ*.

In contrast, COX1/COX2 inhibition did not protect Test Subgroup 3 capillaries from acute Δ*τ*. The negative (not positive) *L*_p_/Δ*τ* relationship with indomethacin (TC2) in this third test subgroup suggested that the natural physiological state of the capillary wall differed and consequently, when COX activity was inhibited the barrier was vulnerable to the flow-induced challenge. The increase in *L*_p_ with indomethacin treatment in Test Subgroup 3 further distinguished this subgroup from the rest, supported hypothesis 1, and reflected the whole organ data reported by Moller and Grandë (1997). Sorting out involvement of COX1 from COX2 and understanding how COX1/COX2 products contribute to mechanotransduction require further study.

### COX1/COX2 inhibition and systemic predictors of capillary *L*_p_

In this study, hypothesis 2 was tested using multiple regression analysis of systemic variables on capillary *L*_p_. The hypothesized prediction of *L*_p_ was not detected for any of the control capillaries. However, indomethacin treatment revealed that RBC or hematocrit, plasma protein, and acute Δ*τ*, accounted for a substantial amount of the variability in *L*_p_ for TC2 in Test Subgroups 1 and 2. The systemic variable used as an index of chronic *τ* (RBC or hematocrit, respectively) differed between Test Subgroups 1 and 2, further suggesting distinct differences between these two subgroups.

Interconnections between chronic *τ* (RBC or hematocrit) and the endothelial glycocalyx (plasma protein) have been demonstrated in the past (Secomb et al. 2001). For example, chronic *τ* thickens the endothelial glycocalyx (Woolf 1982; Wang et al. 1991) and produces changes in its composition (Gouverneur et al. 2006). Furthermore, it has long been known that lowering plasma protein increases permeability (Danielli 1940; Myhre and Steen 1977), a mechanism thought to be loss of protein from the glycocalyx (Schneeberger and Hamelin 1984; Huxley and Curry 1985). Separation between prostacyclin and the glycocalyx also has been demonstrated (Pahakis et al. 2007). To our knowledge the data presented here are the first to demonstrate a relationship between systemic variables and function of living, intact capillaries when assessed in vivo in the same animal.

#### RBC and hematocrit

Warboys et al. (2010) exposed cultured endothelial cells to chronic *τ* for 6 to 8 days and reported decreased permeability to rhodamine albumin, consistent with the negative estimates for RBC in the multiple regression model for Test Subgroup 1. These data are consistent with chronic adaptation of *τ*-sensitive components of the capillary wall, in part, due to blood viscosity-induced *τ*. Hematocrit, which was related linearly to RBC in this study (data not shown), has been well established to be the main source of viscosity in the microcirculation (Lipowsky 2005). Adaptation to the significantly lower RBC in Test Subgroup 2 may have contributed to the high *L*_p_ values for TC1 by a similar mechanism.

In contrast to the results reported here, Warboys et al. did not detect a change in permeability with indomethacin. These conflicting results may be accounted for by different protocols or different assay sensitivity. In this study, intravital microscopy insured that capillaries remained within the tissue and perfused. Each chronic *τ*-adapted capillary was then challenged with acute Δ*τ*. Warboys did not add the acute Δ*τ* stimulus, a distinct difference between the two protocols. Warboys did, however, provide important insight into adaptation of endothelial cells to chronic *τ* and, similarly, the individual capillary method is likely the more sensitive assay for demonstrating permeability responses in vivo. Adamson et al. (2013) reported little impact of chronic *τ* on venular *L*_p_ where the assessments were initiated well past the time courses reported here. Although it is difficult to determine whether the venules studied by Adamson experienced an acute *τ* challenge, the protocol was more akin to that of Warboys and difficult to compare with the one used here.

#### Plasma protein

A second significant factor in the model that predicted capillary *L*_p_ with indomethacin was plasma protein, a systemic variable that has been well established to interact with the glycocalyx (fiber matrix/endothelial surface layer) of the cardiovascular endothelium. All capillaries in this study were devoid of rolling or sticking WBC suggesting that the in vivo glycocalyx was not damaged, but leaving open the possibility that slight differences in plasma protein were impacting *L*_p_ via their interaction with the glycocalyx. Indomethacin treatment revealed both a positive (Test Subgroup 1) and negative (Test Subgroup 2) impact for plasma protein on capillary *L*_p_ (Table3), with the greater impact identified for Test Subgroup 2. This negative relationship suggested that lower plasma protein was associated with higher *L*_p_, a result that is consistent with observations in the past (Danielli 1940; Myhre and Steen 1977). Pahakis et al. (2007) reported that prostacyclin release did not change with digestion of different components from the endothelial glycocalyx suggesting that, here, the COX1/COX2 machinery remained unaffected by the subtle changes in the glycocalyx that might occur across the range of plasma protein found in healthy animals.

**Table 3:** Multiple regression model parameters for predicting capillary hydraulic conductivity (*L*_p_) with indomethacin

Prediction Variable	Model *R*^2^	Model *P*-value	Parameter	Estimate (±SE)	*P*-value
Test Subgroup 1					
Indomethacin *L*_p_	0.59	<0.0001	Δ*τ*	0.081 (±0.021)	0.002
			RBC	−0.000005 (±0.000002)	0.009
			Pl Pr	0.076 (±0.029)	0.02
			Intercept	−0.80 (±1.32)	0.55
Test Subgroup 2					
Indomethacin *L*_p_	0.96	0.002	Δ*τ*	0.078 (±0.016)	0.009
			Hct	0.18 (±0.03)	0.003
			Pl Prot	−0.210 (±0.027)	0.001
			Intercept	0.72 (±0.8)	0.42

Δ*τ*, change in shear stress; RBC, red blood cell concentration; Pl Pr, plasma protein; Hct, hematocrit.

#### Acute Δ*τ*

It is important to note that a relationship between *L*_p_ and Δ*τ* was absent for TC2 in the Control group and Test Subgroups 1 and 2. However, when combined with RBC or hematocrit and plasma protein, Δ*τ* did contribute to the prediction model. As a result, 59% and 96% of the variability in TC2 *L*_p_ was accounted for when indomethacin was present. The model failed to predict *L*_p_ for Control group TC2, further supporting the impact of the drug intervention.

The similar contribution of Δ*τ* in the prediction models (Table3) suggested that the mechanisms for mechano-sensing and -transducing acute Δ*τ* remained unaltered by the protocol and the experimental treatment. If, in fact, the unidentified mechanism for mechanotransduction was not compromised, this unique observation suggests that adaptation to chronic *τ* plus a protein-buoyed glycocalyx may play synergistic rather than individual roles in sensing and transducing acute Δ*τ* in intact, living capillaries.

### Dynamic range of capillary tone

Krogh introduced the concept of capillary tone or “tonus” almost a century ago (Krogh 1922). The experiments used to confirm the Test Subgroup 1 prediction model prospectively illustrated a spectrum for “*L*_p_ tone” in intact, living capillaries. With COX1/COX2 inhibited, a well-organized response to Δ*τ* that was, in part, modulated by RBC or hematocrit and plasma protein became apparent. The *L*_p_/Δ*τ* scatter plot (Fig.5B) for the prospective data provided boundaries for *L*_p_ tone that spanned left and right of the line of identity (Fig.5A). In turn, the Identity data subset overlapped with that from Test Subgroup 1 demonstrating the reproducibility of the original results. In contrast, data from the Control group did not cluster within the boundaries (not shown), suggesting a broader, less controlled response when COX1/COX2 was not inhibited.

#### Crosstalk of COX with other cell signals

The results from this study provide insight into the fact that capillary *L*_p_ responses to Δ*τ* likely depend on the state of intracellular enzyme activity at the time of mechanical stimulation. Studies of cultured endothelial cells indicate that prostacyclin is coreleased with nitric oxide (de Nucci et al. 1988), that crosstalk exists between these two signaling molecules (Osanai et al. 2000), and that their release kinetics vary (Mitchell et al. 2007). Reports also indicate that COX1 is the primary isoform expressed by cultured endothelial cells exposed to chronic *τ* and that prostacyclin is the most prominent product of COX1 in these cells (Potter et al. 2011). Consequently, the data in Figures2 and 5, when interpreted in the context of COX/NOS crosstalk, temporal differences in release of prostacyclin and nitric oxide, and COX1 activity can be extrapolated to support the existence of three states of capillary tone: (1) a quiescent state where COX and NOS products are either playing off each other or in balance (Test Subgroup 1), (2) a state where COX activity is high and NOS activity may be high (Test Subgroup 2), and (3) a state where COX activity is low and NOS activity may be high (Test Subgroup 3).

A balance or imbalance between prostacyclin and nitric oxide production may explain the different results reported on intact capillaries and NOS inhibition (Rumbaut et al. 1995; He et al. 1997). The range of *L*_p_ reported by He et al. was 1.4 to 8.2 × 10^−7^ cm·sec^−1^·cm H_2_O^−1^, which would place them into Test Subgroup 1 (or 3). He et al. reported that NOS inhibition increased *L*_p_, consistent with lower nitric oxide enhancing prostacyclin release in vitro (Osanai et al. 2000). In contrast, one absolute value for *L*_p_ (19.6 × 10^−7^ cm·sec^−1^·cm H_2_O^−1^) reported by Rumbaut et al. (1995) places at least some of their capillaries into Test Subgroup 2 here where higher COX activity suggested a different COX isoform (likely COX2). Elevated nitric oxide via inducible nitric oxide synthase (iNOS) does occur in endothelial cells (Moncada et al. 1991; Cortese-Krott et al. 2014) and crosstalk between COX2 and iNOS products has been reported to enhance COX2 activity (Liu et al. 2009). The Rumbaut result is consistent with decreased COX2 activity secondary to decreased nitric oxide with NOS inhibition.

The data in Figure5 add further insight into *L*_p_ function if it is assumed that those capillaries were in a quiescent state. As such, we speculate that for quiescent capillaries prostacyclin produced by COX1 may buffer or blunt the *L*_p_ response to acute Δ*τ*. If so, the data illustrate a spectrum of capillary *L*_p_ tone where *L*_p_ oscillates, shifting left or right of the line of identity (Fig.5A and B) and most likely depending, in part, on COX and NOS activity. From this reasoning it would be reasonable to anticipate outliers in any *L*_p_/Δ*τ* linear regression analysis, for, in fact, the true geometry of the relationship may be closer to a triangle and if a third axis, time, is added, then a cone shape may be the best graphical representation of fluid movement from a collection of capillaries in response to fluid mechanical force in vivo. Systematic studies in vivo that focus on COX/NOS crosstalk, enzyme isoforms, and flow-induced force are required.

### Summary and implications

Results from studies of cultured endothelial cells suggest that chronic *τ*, the glycocalyx, and acute Δ*τ* would be involved with control of capillary *L*_p_. The unexpected results here were the fact that COX1/COX2 enzyme activity was the intracellular-signaling “curtain” that needed to be drawn in order to view empirically the relationships between system variables and function of capillaries. The integrated, synergistic, and coordinated events that occur when a capillary adjusts to Δ*τ* may be influenced by adaptation to chronic *τ* and interaction of plasma proteins with the glycocalyx. To our knowledge, systemic variables and capillary *L*_p_ have not been associated previously.

Data presented in the study suggest that minute adjustments in tissue hydration may occur in vivo with changes in flow. Maintaining barrier function may be an unrecognized benefit of sustained RBC and protein nutritional status in health as well as replacement of lost RBC and/or protein clinically. If edema is present, increased chronic *τ* and protein may contribute to restoring barrier function. Inhibiting COX1/COX2 may be a less reliable alternative for restoring barrier function depending on the natural balance of enzyme activity in the endothelial cells of the animal or individual.
